# Parasitic findings on threatened pudu deer from Central Chile accounts first genetic characterization of lice parasitizing *P. puda* in Chile and the first molecular report of *Taenia hydatigena* metacestodes in this species

**DOI:** 10.1080/01652176.2024.2302027

**Published:** 2024-01-19

**Authors:** Tamara Muñoz-Caro, María Fernanda González, Rodrigo Villalobos, Alejandro Hidalgo

**Affiliations:** a Escuela de Medicina Veterinaria, Facultad de Medicina Veterinaria y Recursos Naturales, Universidad Santo Tomás, Talca, Chile; b Médico Veterinario Encargado de Fauna Silvestre, Servicio Agrícola Ganadero (SAG), Talca, Chile; c Laboratorio de Inmunoparasitología Molecular, Departamento de Ciencias Preclínicas, Facultad de Medicina, Centro de Excelencia en Medicina Traslacional (CEMT), Universidad de La Frontera, Temuco, Chile

**Keywords:** Southern pudu, *Pudu puda*, *Taenia hydatigena*, lice, louse, wildlife conservation, Chile, threatened species

## Abstract

Southern pudu (*Pudu puda*) is a threatened endemic deer of the temperate forests of Chile. In recent years pudu populations rates have decreased mainly due to anthropogenic causes including forest loss and landscape fragmentation. In this context, the parasitic fauna of Chilean pudu has been scarcely investigated. The aim of this study was to determine the parasitic status of rescued pudu *n* = 13 from its natural habitat in Central Chile (Maule region) during March 2022 and June 2023 by applying morphological, histopathological, and molecular analyses. As result, we report the presence of transmission of parasites from dogs to pudus as showed by the presence of metacestodes of the parasite *Taenia hydatigena* on omentum, liver, and pleura of pudus during postmortem examinations, being the first molecular report on the presence of this parasite on Chilean pudu. Meanwhile, ectoparasite examinations determined the presence of chewing and sucking lice on pudu exemplars here analysed. Molecular and phylogenetic analysis of lice revealed new insights on *Bovicola* and *Anoplura* lice parasitizing *P. puda* in Chile, equally being the first genetic characterization of lice parasitizing pudu exemplars in Chile. In addition, parasite loads of lice and metacestodes were analysed. However, no statistically significance was observed when comparing environmental and individual traits influence on parasite load variation. Overall, the study area is the northern limit of habitat distribution of this specie in Chile and we here provide novel information on pudu deer parasites, thus making a useful and valuable contribution to the parasitological knowledge on this threatened species.

## 1. Introduction

The southern pudu (*Pudu puda*) is an inconspicuous species endemic to the temperate forests of Chile. Is one of the smallest deer in the world (Hershkovitz 1982) and one of the least studied mammals of Chilean forest fauna (Weber and Gonzalez 2003).

According to the IUCN (Silva-Rodríguez et al. 2016), the conservation status of the pudu is Near Threatened with an estimated 10,000 individuals distributed from 36–49°S in Chile (Miller et al. 1973). However, to date, this estimated population size is not supported by quantitative local data estimating its abundance. Thus, in recent years pudu populations have decreased dramatically mainly due to anthropogenic causes including forest loss and landscape fragmentation (Silva-Rodríguez et al. 2010). Likewise, studies have indicated that the habitat of this threatened cervid is poorly represented by the Chilean system of nationally protected areas (Pavez-Fox and Estay 2016) being the more suitable areas in Central Chile highly fragmented and used for agricultural, forestry, or other human activities. Furthermore, investigations on activity patterns of Chilean pudus suggest that this specie adjusts its spatio-temporal ecology as a mechanism to reduce predation risk (Zúñiga and Jiménez 2018). Thus, the pudu is also being affected by predation by feral and domestic dogs, competition with exotic species, and poaching activities (Silva-Rodríguez et al. 2010). However, given their evasive behavior, this specie remains poorly studied in its natural habitat (Pavez-Fox and Estay 2016).

The study of endo- and ecto-parasites of animals may provide novel insights on the interactions and sympatry of wild and domestic animals as well on parasite diversity affected by anthropogenic factors linked to ecological disruption (Daszak et al. 2000). To the authors’ knowledge, the parasitic status of wild Chilean pudu has been investigated, including descriptive reports on protozoans and helminths (Díaz et al. 1977; Lobão-Tello et al. 2017), nonetheless, lacking on further molecular analyses. Therefore, the aim of this work was to determine the parasitic status of *P. puda* in Central Chile (Maule region), the northern limit of habitat distribution of this specie in Chile. The parasitic findings includes molecular analyses identifying taeniid species as well as sucking and chewing lice. As second objective, main causes of rescue and death of pudus will be here also investigated.

## 2. Materials and methods

### 2.1. Study area and parasitological examination

During March 2022 and June 2023, a total of 13 exemplars of southern pudu (*P. puda*) were rescued from its natural habitat within Central Chile in Maule region (35°25′36″S 71°40′18″O) by the Agricultural and Livestock Service (Table 1). Depending on the cause of rescue and health condition, animals were either transported to the University Clinic of Santo Tomás School of Veterinary Medicine in Talca, Chile, or to the Laboratory of Anatomic Pathology of same University in cases of exemplars found dead or who did not survive to acquired injuries. Complete necropsy procedures were conducted on each case as per standard protocol followed in Anatomic Pathology Laboratory of the Santo Tomás University analyzing internal organs in search for endoparasites. Extracted metacestode cysts were detonated discarding the liquid part and the invaginated scolex of each cyst was dissected and transferred into glass bottles containing 70% ethanol according to Kilinc et al. 2019. Accordingly, molecular identification was performed in order to confirm morphological diagnosis as further explained. Tissue sections for histopathological evaluation were fixed in 10% neutral buffered formalin, routinely processed, sectioned at 4 μm, stained with haematoxylin and eosin and examined with a light microscope.

**Table 1.** Summary table of date of rescue, season, sex, age, rescue areas, causes of rescue, rescue status, parasitic findings, and parasite load on examined *Pudu puda* during March 2022 and June 2023 within Maule region (the northern limit of habitat distribution of this specie in Chile).

Date	Season	Sex (Female/Male)	Age (< or > 1 year old)	Rescue area	Cause of rescue	Rescue status	Parasitic findings and location	Parasite load
03-03-2022	Summer	F	< 1	35°05′37.42 S 72° 0105.32 O	Attack by dogs	Alive	Negative	–
10-05-2022	Autumn	M	> 1	35°28′33 S 72°03′33 O	Attack by dogs	Dead	Negative	–
02-06-2022	Autumn	M	> 1	35°31′67 S 72°25′ O	Attack by dogs	Dead	Negative	–
14-07-2022	Winter	F	> 1	35°05′37.42 S 72°01′05.32 O	Attack by dogs	Dead	Lice (fur) *C. tenuicollis* (omenta)	Lice: high (*Bovicola*); low (*Anoplura*) *C. tenuicollis*: low
16-09-2022	Winter	M	> 1	35°1′19 S 72°2′40 O	Attack by dogs	Dead	*C. tenuicollis* (omenta and pleura)	Low
11-10-2022	Spring	M	> 1	35°14′45 S 71°58′54 O	Attack by dogs	Dead	*C. tenuicollis* (omenta)	Low
19-10-2022	Spring	M	> 1	35°04′98.96 S 72°06′79.78 O	Attack by dogs	Dead	Lice (fur) *C. tenuicollis* (liver)	Lice: high (*Bovicola*); low (*Anoplura*) *C. tenuicollis*: low
02-11-2022	Spring	M	> 1	35°05′37.42 S 72°01′05.32 O	Forest fires	Alive	Negative	–
14-11-2022	Spring	F	> 1	35°17′00″S 71°16′00′O	Run over	Dead	Negative	–
16-04-2023	Autumn	F	> 1	35°20′00 S 72°25′00 O	Attack by dogs	Dead	Lice (fur) *C. tenuicollis* (liver)	Lice: high (*Bovicola*); low (*Anoplura*) *C. tenuicollis*: low
17-04-2023	Autumn	M	> 1	35°28′33 S 72°03′33 O	Attack by dogs	Dead	*C. tenuicollis* (omenta)	Low
06-05-2023	Autumn	M	> 1	35°07′20.15 S 72°10′88.59	Attack by dogs	Dead	*C. tenuicollis* (omenta)	Low
16-06-2023	Autumn	F	> 1	35°04′98.96 S 72°06′79.78 O	Attack by dogs	Dead	Lice (fur)	High (*Bovicola*); low (*Anoplura*)

Moreover, pudus were examined in search for ectoparasites by visual examination of the host pelage. Ectoparasites were collected by hand or by using combs and preserved in 70% ethanol. The specimens were then transferred to Amman lactophenol for diaphanization (>24 h) and mounted in glycerinated gelatin for observation as a modification of the method proposed by Philip-Samuel et al. (2021).

Morphological analysis was performed under stereomicroscope (Motic) coupled into a digital camera (CMOS) jointly with a Leica microscope DM750 equipped with a Flexacam C3 camera (18 mpx) by using standard taxonomic reference such as Price et al. (2003) thereby dividing specimens in chewing and sucking lice. After this, specimens were stored at 4 °C for further molecular analysis.

Parasitological analyses were carried out in the Laboratory of Veterinary Parasitology of Santo Tomás School of Veterinary Medicine in Talca, Chile, and in the Laboratory of Molecular Immunoparasitology of the Faculty of Medicine of the Universidad de La Frontera in Temuco, Chile.

In all rescued pudus, data on date of rescue, rescue area, season, sex, age, cause and state of rescue (alive or death), parasitic findings and parasite load were registered. In the present study, recovered pudus were afterward derived into conservation and rehabilitation centers among Maule region. The authors confirm that the ethical policies of the journal, as noted on the journal’s author guidelines page, have been adhered to.

### 2.2. Genomic DNA extraction and PCR amplification of parasites

#### 2.2.1. Lice

For molecular analysis, a pool of lice specimens coming from 3 different animals and from each morphological group (chewing and sucking lice) were individually crushed by sonication in 1.5 ml tubes followed by the extraction method according to Sambrook et al. (1989), modifying the precipitation step with phenol/chloroform by ammonium acetate (pH 8.0)/chloroform. DNA integrity tests were performed by agarose gel electrophoresis and quantification and purity assessment were measure by NanoQuant. Complete sequences of the mitochondrial *cox1* gene and 18S rRNA published in GenBank for various genera of Psocodea (*Ischnocera* and *Anoplura*) were obtained and analysed by multiple alignment using the ClustalW algorithm of the BioEdit program with a boostrap of 1000 comparisons. Then, based on the consensus sequences obtained, primers were designed by using the Primer3Plus program and were further tested in a temperature gradient (50-65 °C) using DNA from *Bovicola bovis* (*Ischnocera*) and *Linognathus setosus* (*Anoplura*) as positive controls respectively. Therefore, the selected primers were *cox1* in *Ischnocera* (forward 5′ GGAGTTGTCTGAAGCGGGTA and reverse 5′ ACCGGAAGAGACAGAAGCAA-3′’; 487 bp), and 18S rRNA for *Anoplura* (forward 5’GGTGAAATTCTTGGATCGTCGC-3′’and reverse 5’CCAGACAAATCGCTCCACCA-3′’; 410 bp). The reaction mix for all PCRs were adjusted to a total volume of 20 µl per sample by using Go Taq®Green MasterMix 2X (10 µl), 10 µM of each primer (1 µl) and nuclease-free water (7 µl). In addition, on each reaction, 1 µl of DNA (>10 ng/µl) was used. For the amplification, the followed protocol was performed: a first step of 5 min at 90 °C was applied, followed by 40 cycles of 45 sec at 95 °C, 60 °C and 72 °C. Finally, a step of 5 min at 72 °C was undergone for subsequent conservation at 4 °C until electrophoresis analysis.

#### 2.2.2. Metacestodes

From collected metacestodes, approximately 5 mm^3^ of each one was transferred into 1.5 ml tubes and macerated with sterile hypodermic needles. DNA extraction was also performed as described for lice. For PCR amplification, the same reaction mix previously described was used but in this case using the following primers: JB3 (5’TTTTTTGGGCATCCTGAGGTTTAT-3′’) and JB4.5 (5’TAAAGAAAGAACATAATGAAAATG-3′’) jointly with the protocol of cycles and temperature described by Bowles et al. (1992). PCR analysis was performed by amplification of *cox1* fragment of 445 bp equal for cestodes, then fragments were sequenced in order to compare the identity of samples with other known sequences published in GenBank.

### 2.3. Phylogenetic analysis

The amplified PCR products of both the lice and metacestode samples were run by electrophoresis (125 V/225mAmp) in 1.8% agarose gels for 45 min and visualized using a transilluminator. The products obtained from each sample (10 µl) were sent for purification and Sanger-type capillary sequencing to an external company (Macrogen). Sequence analysis was performed by multiple alignment (ClustalW, BioEdit) and comparison on the GenBank database through the BLAST tool (Basic Local Alignment Search Tool). The phylogenetic reconstruction analysis for both species of lice was inferred by the Maximum Likelihood method, with a boostrap of 1000 replications and Gamma distribution of 3 discrete categories. In the phylogenetic analysis of Ischnocera, the 3-parameter Tamura model was applied (Tamura 1992). In the Anoplura, the Kimura model of 2 parameters was applied (Kimura 1980). Initial tree(s) for the heuristic search were obtained automatically by applying Neighbor-Join and BioNJ algorithms to a matrix of pairwise distances estimated using the Maximum Composite Likelihood (MCL) approach. The rate variation model allowed for some sites to be evolutionarily invariant (42.62% sites). These analyses were performed in Mega X software.

Meanwhile, the criteria for sequence selection were based on search of partial sequences of ribosomal or mitochondrial genes used in phylogeny (18S rRNA and cox1 respectively), with available information on GenBank, which could be aligned to our regions of genes analysed, and likewise presenting diversity of variable sites. Accordingly, those sequences that could not be compared were discarded, mainly due to incomplete information or because did not align with the regions of the genes analyzed.

### 2.4. Statistical analyses

Statistical analyses were performed following Morrison (2002) suggestions. Fisher exact test was applied in order to calculate P values searching for statistical associations between season (autumn, winter, spring, summer), sex (male/female), age (< 1 year old/> 1 year old), rescue state (dead/alive), parasitic findings: metacestodes and lice. Parasite load (low, medium and high) was compared by using non-parametric Kruskal-Wallis test. Defining low, when parasitic findings ranged between 0 and 5; medium, between 6 and 10, and high when more than 11 parasites were found. The significance level was set at *p* < 0.05.

## 3. Results

### 3.1. Ectoparasites found on examined Pudu puda

**Figure 1.**
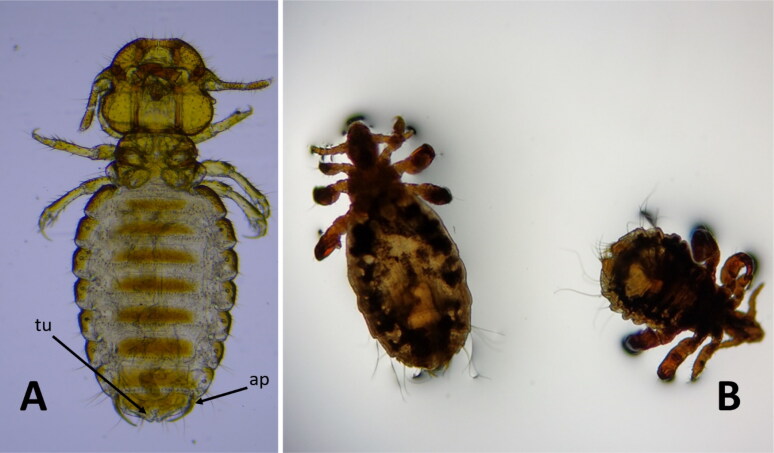
Lice found on fur on examined pudus. A: Adult female of genera chewing louse. tu: terminal tubercles; ap: lateral appendages. B: Adult and third nymph stage exemplars of sucking louse.

As result of external examination in search for ectoparasites on fur of rescued pudus, only lice specimens were found coming from 4 different examined pudus. Morphological examination of lice resulted in the identification of chewing and sucking lice, observing not only adult stages but also nymphs as shows Figure 1.

**Figure 2.**
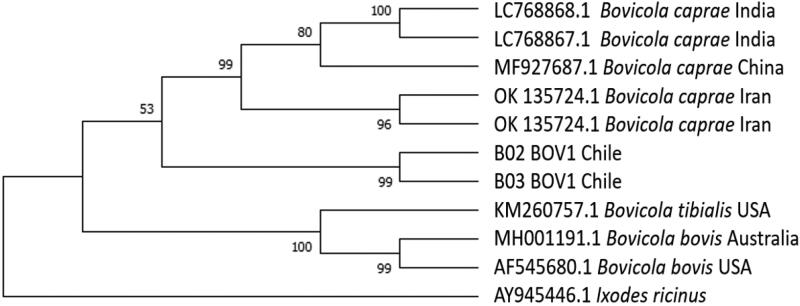
Evolutionary analysis of *cox1* gene from *Ischnocera* lice by Maximum Likelihood method. B02 and B03 are sequences obtained from lice of *Pudu puda*.

Morphological analysis of chewing lice was performed according to taxonomic keys (Emerson and Price 1975). In the present study, the structure of the genitalia in female chewing lice showed two prominent terminal tubercles and two lateral elongated and sharp appendages reaching the terminal tubercles (Figure 1).

Moreover, the morphological analysis of the sucking lice could not be achieved since the few specimens collected were prioritized for DNA extraction and further molecular assays.

Parasite loads of lice were analysed, being high (more than 11 specimens) for chewing lice, and low (1 to 5 specimens) for sucking lice. Nonetheless, when comparing total lice infestation and parasite loads according to chewing and sucking lice to individual factors such as sex, age, season and rescue status (dead or alive), no statistically significance was observed (*p* > 0.05 in all cases).

PCR amplification was successful with the primers designed for the *cox 1* fragment in *Ischnocera* lice and for *Anoplura* lice where a fragment of the 18S rRNA was amplified and compared with available sequences on the databases.

The phylogenetic analysis of the genus *Bovicola* belonging to *Ischnocera* lice showed that the sequences of the analysed samples (B02 and B03) are closely related to the species cluster of *B. caprae* isolations and more separated from other species of the genus such as *B. bovis* and *B. tibialis (Figure 2)*.

**Figure 3.**
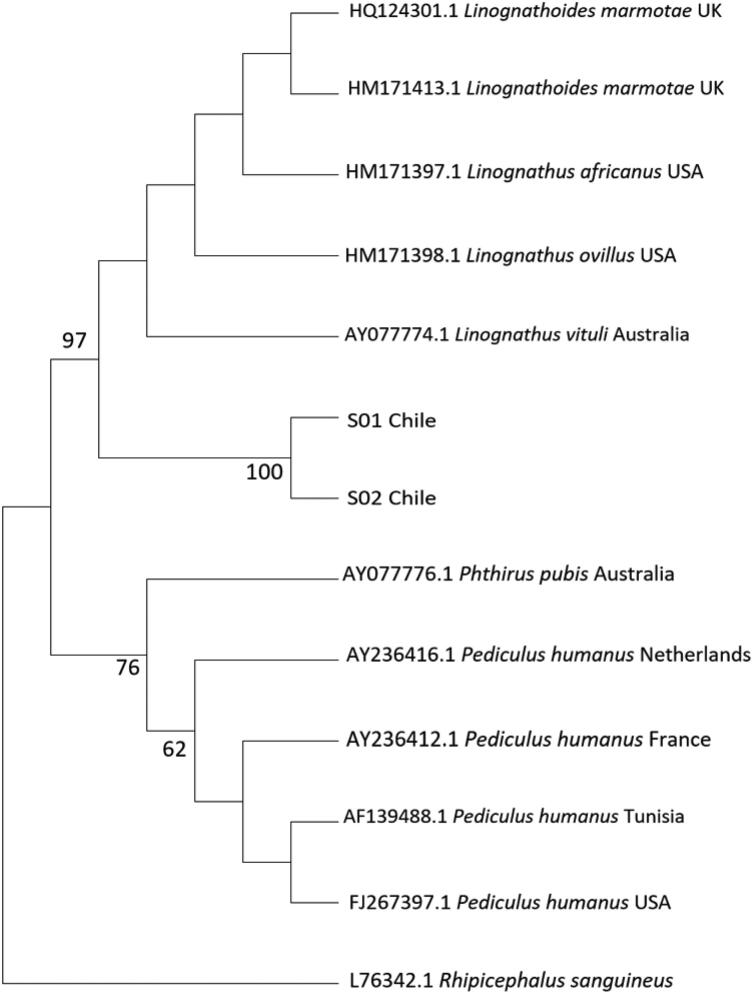
Evolutionary analysis of 18S rRNA gene from *Anoplura* lice by Maximum Likelihood method. S01 and S02 are sequences obtained from lice of *Pudu puda.*

Similarly, the analysis of potential *Anoplura* candidates showed the divergence with *Linognathus* from a common ancestral node with a high support (97%), then both branches in turn diverge in an expected way with the *Pediculidae* group (Figure 3).

### 3.2. Endoparasites found on necropsied Pudu puda

**Figure 4.**
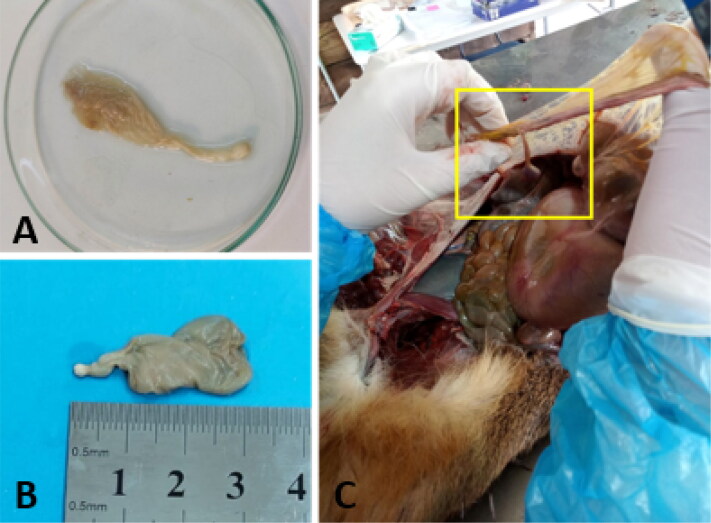
Metacestode of *Taenia hydatigena* (*Cysticercus tenuicollis*) found within abdominal cavity of *pudu puda*. **A** and **B**: findings of metacestodes dissected out from omenta within abdominal cavity. **C**: metacestode *in situ* attached to greater omentum.

**Figure 5.**
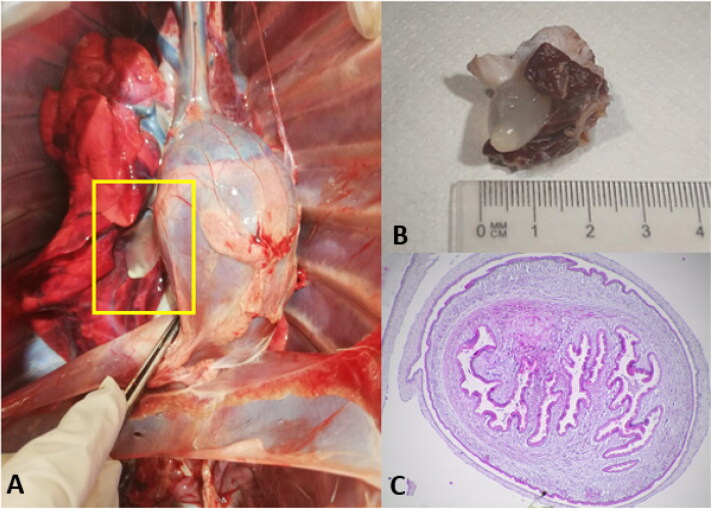
Ectopic localization of *Cysticercus tenuicollis* metacestode within thoracic cavity. **A**: metacestode *in situ* attached to visceral pleura. **B**: metacestode dissected out from pleura showing invaginated scolex. **C**: histological section of the scolex showing suckers (protoscolex) and outer membrane by using hematoxylin-eosin staining 400X magnification.

Postmortem examination was performed on 11 rescued pudus from Central Chile (Maule region) who did not survive to acquired injuries. Interestingly, in 7 cases, metacestodes compatible with *Cysticercus tenuicollis*, the larval stage of *T. hydatigena*, were identified based on morphological features (Figures 4 and 5). These were found within abdominal cavity in omenta (greater omentum, Figure 4), liver and in ectopic localization on pleura (visceral) within thoracic cavity (Figure 5). Histological section of a larva (scolex) of the ectopic cyst of *C. tenuicollis* was performed by using hematoxylin-eosin staining observing suckers (protoscolex) of larva and connective tissue as indicated in Figure 5. We did not observed features of severe inflammatory infiltration associated with the parasite *in situ* neither on omenta (macroscopically) nor in pleura based on histopathological analyses. Parasite load was individually analysed resulting in low infections with 1-5 metacestodes found on each infected animal. However, when comparing total parasite and parasite loads to individual factors such as sex, age, season and rescue status (dead or alive), no statistically significance was observed (*p* > 0.05 in all cases).

In addition, PCR analysis of metacestodes found on abdominal cavity and pleura confirmed the parasite *T. hydatigena* on pudus. As outcome, the molecular analysis of the metacestodes achieved the identification of *T. hydatigena*, since the *cox1* barcode fragment showed 99% and 100% identity with the sequences published in GenBank.

### 3.4. Main causes of death of rescued Pudu puda

Diverse causes of death were reported on rescued pudus including attack by dogs, forest fires and run overs as indicate Table 1. The main cause of death was noticeably the attack by dogs, reported in 10 of 11 examined pudu exemplars.

## 4. Discussion

Forest loss provoked by the accelerated increase of human activities and land uses in areas originally covered by native forest in Chile have been described as main threats for *P. puda* conservation along years (Jiménez and Ramilo 2013). In the present study, we provide evidence on the negative effect of close interaction between *P. puda* and dogs, being the attack by feral and domestic dogs the main cause of rescue and death of the pudu exemplars here analysed. This issue could be associated with the finding of metacestodes of *T. hydatigena* on omenta, liver and pleura during postmortem examinations, thereby proving the occurrence of *T. hydatigena* lifecycle and transmission from dogs or other potential hosts such as foxes and cats to wild pudus populations of Central Chile. These findings are relevant especially considering the lack of updated prevalence data of *T. hydatigena* on other susceptible hosts such as sheep, goats and cattle within the country. Adult stages of the parasite have been recorded only from domestic dogs (Alcaino and Gorman 1999) and in small intestine of South American foxes *Lycalopex culpaeus* and *Lycalopex griseus* (Oyarzún-Ruiz et al. 2020). *Taenia hydatigena* is a cosmopolitan parasite encountered in dogs and wild carnivores in its mature state, and whose larvae, *C. tenuicollis* is seen on ruminants and pigs (Soulsby 1982) which are intermediate hosts. Clinical signs on intermediary hosts infected with *T. hydatigena* vary according to the severity of infection (Christodoulopoulos et al. 2008). However, in heavy infections, the migrating larvae could produce intensive destruction of internal organs with eosinophilic infiltration and severe inflammation that could be fatal (Bamorovat et al. 2014; Radfar et al. 2014). In the examined pudus of this study, metacestodes of *T. hydatigena* were found in low parasitic load mainly in great omentum, liver and in ectopic localization in pleura with no evidence of severe inflammatory reactions induced by the parasite, as seen both, macroscopically and under histological analyses. Based on this, these results state that there is no evidence that this taeniid is a health (nor conservation) concern in the case of pudu. Nonetheless, we postulate that higher parasitic loads could provoke pathological manifestations specially on pudus subjected to prolonged stress, immunosuppression, or other concomitants pathologies as observed for other intermediate hosts affected by cysticercosis such as lamb and sheep (Scala et al. 2014, 2015). In addition, when metacestodes cause injure, the likelihood of being transmitted to the definitive host increases along with the accomplishment of the parasite life cycle (Corda et al. 2020). In the present study, the influence of sex, age, season and rescue status (dead or alive) on parasite load was statistically analysed resulting in no significant differences, being these findings in accordance with other studies analysing influence of sex, age and host breed on the prevalence of *C. tenuicollis* in sheep (Senlik 2008) where only significant differences on host breed were found. However, in case of lice, studies have shown the influence of sex and seasonality on parasite load (Fernandes et al. 2012; Duboscq et al. 2016) contrary to our results, probably due to the small sample size considering working with an endangered species.

Remarkably, this is the first genetic characterization of lice parasitizing *P. puda* in Chile. Phylogenetic analysis of chewing lice revealed that the sequences of the samples analyzed are closely related to the cluster species *B. caprae.* In addition, in morphological analyzes, some features observed in the structure of the genitalia in female chewing lice showed differences with descriptions for *B. caprae*: two prominent terminal tubercles are seen absent in *B. caprae* in which only a V-shaped terminal invagination was observed. In addition, the lateral appendages are more elongated and sharper, reaching the terminal tubercles unlike *B. caprae.* Thereby, we here provide new insights on morphological aspects of *Bovicola* species parasitizing *P. puda* in Chile. However, further research is required to suggest that this could be a potential new species. Thus, *B. caprae* has been identified in pudus and caprine from Chile (González-Acuña et al. 2004, 2005), nonetheless these data were merely descriptive and not supported by any molecular analyses. In line, based on the evasive and solitary behavior of *P. puda* a direct transmission route of lice from domestic ruminants is unlikely to occur. However, the potential role of fomites as an alternative indirect route of transmission should be accounted considering that pudus may share their environment with domestic species.

Moreover, the analysis of the samples of sucking lice showed with a high support (97%) the divergence with genus *Linognathus* and with the *Pediculidae* group. Although a detailed morpholo­gical analysis was not possible to achieve due to few specimens obtained considering working with threatened species, it was possible to describe that the rostral portion of the head of the specimens were blunt, a characteristic consistent with the genus *Solenopotes*, which differs with the morphology of the heads of the genera *Linognathus* and *Haematopinus* also present in artiodactyls. This suggests that specimens analyzed could be *S. bipinulosus* described in pudus by González-Acuña et al. (2004). However, only two partial sequences of the 18S rRNA gene of *S. capillatus* are available in GenBank and both fragments do not match the fragment analyzed in this study since they are further away towards the 3′ end. Thus, it should be considered the fact that ribosomal genes such as 18S rRNA are highly conserved in most species and especially in insects where a faster substitution rate is observed in mitochondrial genes, which explains why the divergences shown in the analyzes are evolutionarily consistent (Kjer 2004). Nonetheless, further molecular, and phylogenetic analyses will provide more information on this matter as well as the possible role of these insects as vectors of diseases in pudus from Chile.

## 5. Conclusions

Our study constitutes the first report on the molecular identification of *T. hydatigena* parasite on pudus in Chile, thereby demonstrating the occurrence of its lifecycle and transmission from dogs or other potential hosts such as foxes to wildlife pudus populations in Central Chile. This is consistent with the fact that the most common cause of death on pudus reported on this study was the attack by dogs, which may be consequence of habitat fragmentation of this threatened cervid. We also provide first genetic characterization of lice parasitizing *P. puda* in Chile. Potential vector-borne diseases transmitted by these ectoparasites on pudus must be addressed in future studies. Thus, the study area is the northern limit of habitat distribution of this specie in Chile. However, despite the present results, the parasitic status found did not allow us to determine the effects on health status in this species and whether conservation actions targeting to control the impact of the studied parasites are required. Nevertheless, although further research is needed, our results provide novel information on pudu deer parasites, thus making a useful and valuable contribution to the parasitological knowledge on this threatened species.
